# Bridging Diagnostics: Evaluating the NG-CARBA-5 Test and PCR Among Carbapenem-Resistant Enterobacterales Isolated from a Multidisciplinary Hospital in Malaysia

**DOI:** 10.21315/mjms-12-2025-888

**Published:** 2026-04-30

**Authors:** Vityashri Parameswaran, Narcisse Mary Sither Joseph, Syafinaz Amin Nordin, Norlaila Sallehhudin, Rosni Ibrahim

**Affiliations:** 1 Department of Medical Microbiology, Faculty of Medicine and Health Sciences, Universiti Putra Malaysia, Serdang, Selangor, Malaysia; 2 Department of Medical Microbiology, Hospital Sultan Abdul Aziz Shah, Universiti Putra Malaysia, Serdang, Selangor, Malaysia

**Keywords:** carbapenemase, rapid detection, Enterobacterales, sensitivity, specificity

## Abstract

**Background:**

Carbapenem-resistant Enterobacterales (CRE) pose a major health threat worldwide because of limited treatment options and high mortality rates. This study investigated the detection of CRE in a multidisciplinary hospital by comparing the multiplex polymerase chain reaction (PCR) with the rapid NG-CARBA-5 assay.

**Methods:**

A total of 58 bacterial isolates, previously recovered from various clinical specimens, were tested. Genotypic identification was performed using a multiplex PCR assay targeting carbapenemases, including *bla*KPC, *bla*NDM, and *bla*OXA-48. The rapid assay was conducted using the NG-CARBA-5 test kit to detect *bla*NDM, *bla*IMP, *bla*VIM, *bla*OXA-48, and *bla*KPC.

**Results:**

Out of the 58 isolates tested, 36 were identified as carbapenemase producers with the rapid assay, whereas 39 were detected by PCR. All 36 carbapenemase-positive isolates detected by the rapid assay harboured the *bla*NDM gene, while multiplex PCR detected 19 isolates with multigenes (18 *bla*NDM+*bla*KPC and 1 *bla*NDM+*bla*OXA) and 20 isolates with single genes (18 *bla*NDM, 1 *bla*OXA, and 1 *bla*KPC). Discrepancies were observed among the detection methods, with four isolates reported as false negatives (FNs). This indicates a possible risk of missing colonised or infected patients, which is important in settings such as intensive care units or outbreaks.

**Conclusion:**

The NG-CARBA-5 test is suitable for rapid initial screening, particularly when complemented by another phenotypic test such as the modified carbapenem inactivation method (mCIM) test.

## Introduction

Many hospitalisations and deaths from bacterial infections worldwide are attributable to antimicrobial resistance. In addition, gram-negative bacteria are the primary cause of multidrug resistance (MDR). Enterobacterales are facultative gram-negative bacteria that cause a wide range of deadly infections, including septicaemia, community, hospital, and ventilator-acquired infections, complex urinary tract infections, intra-abdominal infections, pneumonia, bloodstream infections, and skin and soft tissue infections (1). Patients diagnosed with carbapenem-resistant Enterobacterales (CRE) infections are challenging to treat as the bacteria become resistant to carbapenems, the antimicrobials that are often used for MDR pathogens (2). Hence, CRE, as described by the World Health Organization (WHO), is one of the most alarming public health threats, and there is a high demand for new therapeutics (3).

Resistance may result from the production of carbapenemases, mutations in the outer membrane proteins, overexpression of efflux pumps, alterations in penicillin-binding proteins, and changes in biofilm components. Carbapenemases are enzymes that hydrolyse carbapenems and other β-lactams and are categorised into three main classes, each with different mechanisms of resistance. Class A includes *Klebsiella pneumoniae* carbapenemase, Class B includes metallo-β-lactamases, and Class D includes oxacillinases (OXAs). The effective and reliable laboratory detection and identification of CRE is essential for the development and implementation of CRE infection control and management strategies. Resistance can be analysed using various methodologies to inform drug selection when strains exhibit carbapenem resistance in clinical settings. However, the complexity of mechanisms that confer bacterial resistance and inconsistent expression of CRE resistance genes complicate the detection of CRE. Therefore, antimicrobial susceptibility testing, biochemical testing, such as the Carbapenemase Nordmann-Poirel test (Carba NP), and molecular testing, including polymerase chain reaction (PCR), are necessary for the comprehensive assessment of CRE to produce reliable outcomes (1, 3).

Countries such as Indonesia and Myanmar reported the highest CRE rates among Asian countries (3). Furthermore, in Malaysia, the New Delhi metallo-β-lactamase (NDM) gene is the most prevalent, followed by the OXA gene, and these two genes are also found in combination, which is a worrying finding (4). This present study sought to determine the presence of carbapenem-resistant genes in samples isolated from hospitalised patients using phenotypic and genotypic methods.

## Methods

### Bacterial Isolate Collection

A total of 58 nonduplicate isolates were retrospectively retrieved from archived specimens. The samples included *K. pneumoniae*, *Escherichia coli*, and *Enterobacter* spp. from the bacteriology laboratory in a multidisciplinary hospital in Selangor, Malaysia. The isolates were not randomly collected; instead, all available eligible isolates obtained within 12 months were included. These bacteria were isolated from blood (*n* = 13), urine (*n* = 14), rectal swab (*n* = 16), pleural fluid (*n* = 1), wound (*n* = 1), tracheal aspirate (TASP) (*n* = 11), tissue (*n* = 1), and bone (*n* = 1) from hospitalised patients. The identities of the bacterial isolates were confirmed using matrix-assisted laser desorption/ionisation time-of-flight (MALDI-TOF).

### Antimicrobial Susceptibility Test

The disk diffusion method on Mueller-Hinton agar was used to determine susceptibility according to the Clinical and Laboratory Standards Institute (CLSI) guidelines. All isolates that exhibited resistance to carbapenem antibiotics (i.e., imipenem, meropenem, and ertapenem) were confirmed by performing an E-test to determine the minimum inhibitory concentration (MIC) of the tested carbapenem antibiotics. The antimicrobial susceptibility test results were interpreted based on the CLSI guidelines. A sensitive strain of *E. coli*, ATCC 25922, was used as a negative control.

### Modified Carbapenem Inactivation Method (mCIM) Test

Carbapenemase production was determined using the modified carbapenem inactivation method (mCIM) according to the CLSI guidelines. Each sample was inoculated into a tube containing trypticase soy broth and a meropenem disk. After incubation for 4 h, the disk was removed and placed onto a Mueller-Hinton agar plate, inoculated with the *E. coli* ATCC 25922 indicator strain. The mCIM yielded a positive result when an inhibition zone around the disk was absent.

### Rapid Detection Test

NG-CARBA-5 (NG Biotech Laboratoires, Guipry-Messac, France) was used to detect genes encoding carbapenemases in Enterobacterales. The clinical samples were inoculated onto blood agar and incubated at 37°C for 18 h. In a microtube, three colonies from an overnight culture were mixed with five drops of extraction buffer. The tube was vortexed and kept aside for 10 min. Thereafter, 100 μL of the mixture was withdrawn and applied to the well of the test card. The result was read after 15 min and interpreted according to the manufacturer’s instructions.

### Genomic DNA Extraction

The isolates were cultured on blood agar plates overnight at 37°C. Single colonies of carbapenem-resistant isolates were cultured in 2 mL of Luria Broth in centrifuge tubes overnight at 37°C. After incubation, the bacterial suspension was centrifuged at 14,000 rpm for 1 min to pellet the bacterial cells. The DNA from each isolate was obtained using the FastPure Bacteria DNA Isolation Mini Kit (Vazyme, Nanjing, China) according to the manufacturer’s instructions.

### Molecular Analysis of Carbapenemase Genes

Multiplex PCR was used to identify carbapenemase genes using primers targeting *bla*KPC, *bla*NDM, and *bla*OXA-48. The primers were obtained from GenScript (NJ, US) (Table 1). The multiplex PCR assay was performed using Multiplex PCR 5× Master Mix (New England Biolabs, USA). Each PCR reaction consisted of 5 μL of Multiplex PCR 5× Master Mix, 1 μL of each 0.5 μM primer, 1 μL of DNA sample, and 8 μL of nuclease-free water. The following amplification conditions were used: initial denaturation at 95°C for 1 min; 30 cycles of 95°C for 20 s, 51°C for 1 min and 68°C for 30 s; and final extension of 68°C for 5 min. The PCR products were subjected to gel electrophoresis to visualise bands corresponding to the respective amplicon sizes. The 3% agarose gel was stained with 0.8 μL of ViSafe Green Gel Stain (Vivantis, Malaysia) and run for 60 min at 60 V. The PCR products were visualised using a UV transilluminator. The PCR was performed in triplicate, and faint bands were analysed with single-plex PCR to confirm their presence.

### Statistical Analysis

Prevalence was calculated based on the mCIM result using the following equation:


$$\text{Prevalence}=\frac{\text{Number of carbapenemase}-\text{producing isolates}}{\text{Total CRE isolates}}\times 100$$

The NG-CARBA-5 test and PCR were compared in terms of sensitivity and specificity. The performance of the rapid test was evaluated against that of PCR, which was considered the reference standard. A 2 × 2 contingency table was constructed to classify the test results into true positives (TPs), false positives (FPs), true negatives (TNs), and false negatives (FNs). Sensitivity was calculated as the proportion of PCR-positive cases correctly identified by the rapid test [TP/(TP+FN)] × 100, and specificity as the proportion of PCR-negative cases that were correctly identified [TN/(TN+FP)] × 100. The positive predictive value (PPV) and negative predictive value (NPV) were also determined.

## Results

### Sample Distribution

Among the 58 Enterobacterales isolates, 84.5% were *K. pneumoniae* (*n* = 49), 6.9% were *E. coli* (*n* = 4) and *Enterobacter cloacae* (*n* = 4), and only 1.7% were *Enterobacter kobei* (*n* = 1). The majority of the samples were isolated from rectal swabs (27.6%), followed by urine (24.1%), blood (22.4%), and TASP (19.1%), while pleural fluid, bone, tissue, and wound each only accounted for 1.7%. Figure 1 shows the distribution of Enterobacterales across clinical samples. *K. pneumoniae* was prevalent in urine samples, whereas multiple bacterial species were discovered in the rectal swabs.

### Rapid Detection Test

A sample was interpreted as positive when one red line appeared in the control region labelled C and one or more lines appeared in the test regions labelled K (*bla*KPC), O (*bla*OXA-48), V (*bla*VIM), I (*bla*IMP), and N (*bla*NDM). A weak line was also considered positive, as the concentration of carbapenemases in the sample affects the line intensity. A sample was interpreted as negative when only one line appeared in the control region. The NG-CARBA-5 test revealed that 62.1% (*n* = 36) of the samples were positive for carbapenemase activity, whereas the remainder did not contain any of the indicated carbapenemases. All positive samples were confirmed to carry the *bla*NDM gene.

### Analysis of Multiplex PCR

Approximately 67.2% (*n* = 39) of the clinical samples harboured at least one carbapenem-resistant gene, and 32.8% (*n* = 19) were negative for the tested genes. Of the 39 carbapenemase-positive isolates detected by multiplex PCR, 19 were positive for two genes (18 NDM + KPC and 1 NDM + OXA-48), 18 isolates harboured the NDM gene, 1 carried the KPC gene, and 1 isolate harboured the OXA-48 gene. The distribution of genes detected using these two methods is shown in Table 2.

### Statistical Analysis

Of the 58 isolates included in the study, 43 were confirmed as carbapenemase-producing isolates. This corresponds to a prevalence of 74.1% among the collected isolates. In the NG-CARBA-5 test, the *bla*NDM gene was detected in 36 (62.1%) CRE, whereas a total of 39 (67.2%) isolates were positive for carbapenemase genes by multiplex PCR. Among the 22 samples that were negative by the NG-CARBA-5 test, four tested positive with multiplex PCR and were thus reported as FNs. Of 39 carbapenemase-positive isolates detected by multiplex PCR, 19 were positive for two genes. In the NG-CARBA-5 test, only single genes were detected. Table 3 shows the classification of the test results as TP, FP, TN, and FN.

Using PCR as the reference method, the NG-CARBA-5 rapid test demonstrated a sensitivity of 89.7%, a specificity of 94.7%, a PPV of 97.2% and an NPV of 81.8%. An NPV of 81.8% indicates that most negative rapid test results are correct, but approximately 18.2% of negative rapid test results are probably FNs (PCR-positive despite a negative rapid test). Therefore, a negative NG-CARBA-5 test result could still be positive by PCR and should therefore be interpreted cautiously for screening purposes.

## Discussion

The widespread use of broad-spectrum antibiotics and the presence of plasmids encoding MDR genes in pathogenic organisms are the main drivers of MDR emergence. The CREs are the most important resistance threats worldwide, according to the Centers for Disease Control and Prevention (CDC) classification of antimicrobial-resistant pathogens (2). Increased morbidity and mortality in nosocomial and community settings have been linked to the rise of CRE. Carbapenemases are a broad class of enzymes that are found in gram-negative bacteria, including Enterobacterales, that provide resistance to carbapenems. Ambler Class A (*bla*KPC), Class B (*bla*IMP, *bla*VIM, and *bla*NDM-1), and Class D (*bla*OXA-48) are the probable classifications for the carbapenemases found in *Enterobacteriaceae*. Many carbapenemase-encoding genes are found on plasmids or other mobile genetic elements, such as integrons and transposons, which greatly facilitate their dissemination (7).

*K. pneumoniae* was primarily identified among the collected samples because it is a common CRE (8). *K. pneumoniae* is a well-established member of Enterobacterales with the ability to colonise mucosal surfaces and persist in hospital settings; therefore, it is frequently detected. Rectal swabs were the most common sample type, followed by urine and blood, which correspond to the common sites of *Enterobacterales* colonisation and infection (9).

Of the 58 CRE isolates, 36 (62.1%) were positive by the NG-CARBA-5 test, all of which harboured the *bla*NDM gene. A total of 39 (67.2%) isolates were positive for carbapenemase genes by multiplex PCR. Among the 22 samples that were negative by the NG-CARBA-5 test, four were positive by multiplex PCR and were reported as FNs. Among these, one isolate was *E. cloacae* that was positive for the NDM gene (mCIM negative and low MIC value), one was *K. pneumoniae* positive for the NDM gene (mCIM negative and low MIC value), one was KPC-producing *K. pneumoniae*, and one was an OXA-producing *K. pneumoniae* with a low MIC.

Compared with PCR as the reference method, the NG-CARBA-5 test showed high sensitivity (89.7%) and specificity (94.7%), with a PPV of 97.2% and an NPV of 81.8%. This indicates its potential as a reliable initial screening tool. Positive NG-CARBA-5 rapid test results are generally reliable, but some negative results (*n* = 22) may still be positive by PCR. In other words, most negative results are correct; however, a small number of cases may be missed. Notable discrepancies were observed between the rapid test and genotypic results. For instance, some samples tested positive for carbapenemase genes via PCR despite being reported as negative in the rapid test, and vice versa. These inconsistencies may be attributed to factors such as low gene expression, gene variants that are not detected by the rapid assay, or limitations in the test sensitivity.

In addition, such FNs may have serious consequences for patient care and infection control in clinical settings. Undetected carbapenemase-producing Enterobacterales can facilitate silent transmission in hospitals, potentially leading to early cessation or inadequate isolation measures (10). This emphasises the importance of confirmatory genotypic testing in negative cases. Specifically, combinations such as *bla*NDM+*bla*KPC or *bla*NDM+*bla*OXA-48 were only detected by PCR, thus demonstrating its superior capacity to identify multigene resistance patterns. However, in one case, a sample tested positive on the rapid test but negative on PCR, which suggests either limitations in primer sensitivity or the presence of genetic mutations. Plus, NG-CARBA-5 detects *bla*KPC and *bla*OXA-48 poorly, unlike PCR, which detects all carbapenemase types precisely. Although PCR requires specific primers, it is more sensitive than the rapid test (11).

A total of 35 samples were confirmed as TPs and 18 as TNs for carbapenemase production, thus indicating the presence of carbapenemase-mediated resistance among the bacterial isolates. Their role is to inactivate β-lactam antibiotics, primarily carbapenems. The capacity of these enzymes to inactivate a wide variety of β-lactams, such as carbapenems and extended-spectrum cephalosporins, is their distinguishing feature. KPC-producing *K. pneumoniae*, especially the globally dominant ST258 strain, and KPC, NDM, or OXA-48-producing *E. coli* ST131 strains are major contributors to the spread of carbapenem resistance (12). Samples with negative results may exhibit different resistance mechanisms, including alterations in outer membrane proteins, overexpression of efflux pumps, and mutations in penicillin-binding proteins and biofilm elements (3).

Furthermore, PCR showed a superior capacity to identify multiple genes, detecting combinations of resistance genes such as *bla*NDM+*bla*KPC and *bla*NDM+*bla*OXA-48, whereas NG-CARBA-5 detected only single genes. NG-CARBA-5 is valuable for the rapid confirmation of CRE, but it is not sufficient as the sole test to exclude infection in high-risk patients or during outbreaks. Therefore, negative NG-CARBA-5 results should be confirmed by PCR or supported by other screening tests such as mCIM. The presence of multiple carbapenemase genes within a single clinical isolate of Enterobacterales highlights the complex genetic basis of antimicrobial resistance and poses serious challenges for detection and treatment. In this study, genotypic analysis using multiplex PCR revealed that many isolates harboured combinations such as *bla*NDM+*bla*KPC or *bla*NDM+*bla*OXA-48, which were not detected by rapid immunoassay alone. This genetic cooccurrence, particularly in *K. pneumoniae*, indicates a heightened potential for horizontal gene transfer and MDR. Such findings are consistent with recent surveillance data in Malaysia (4), where the prevalence of multigene combinations, especially involving *bla*NDM, is a growing concern.

Despite its insightful conclusion, this study has several limitations. First, the comparatively small sample size (*n* = 58) limits the statistical power and the generalisability of the findings to larger groups (13). It is also challenging to extend the results to other Enterobacterales species due to sample bias arising from the predominance of *K. pneumoniae* isolates (14). Furthermore, only three panels of carbapenemase genes were targeted by the multiplex PCR in this study, which may have resulted in an underestimation of resistance by excluding other notable genes such as *bla*IMP and *bla*VIM, which are commonly included in broader multiplex PCR panels used for carbapenemase detection (15). Although the NG-CARBA-5 test performed well overall, its limitations as a standalone diagnostic tool are emphasised by FNs and its inability to identify multiple carbapenemase genes in co-producing isolates (16). Finally, the results may not be fully generalisable across different clinical settings due to the imbalance in sample types and the single-centre setting. Therefore, it is recommended that these findings be validated and expanded through larger, multicentre investigations with expanded molecular panels.

## Conclusion

In conclusion, the NG-CARBA-5 test provides rapid and specific CRE detection, which makes it a valuable tool for early confirmation. However, its lower NPV means that some cases may be missed, especially if the bacteria produce carbapenemases that the test does not detect or are present in low amounts. Therefore, in high-risk patients or during outbreaks, negative results should be interpreted cautiously and confirmed by PCR to ensure accurate detection and prevent unrecognised transmission. Another limitation observed in NG-CARBA-5 is that no multiple genes were detected. However, this information is useful for surveillance and epidemiological data. NG-CARBA-5 can be a beneficial initial diagnostic tool in addition to other phenotypic methods, such as susceptibility testing, mCIM, and EDTA-modified carbapenem inactivation method (eCIM). Thus, in healthcare settings, a combined diagnostic approach improves surveillance accuracy, guides targeted treatment, and facilitates the prompt containment of resistant strains.

## Figures and Tables

**Figure 1:**
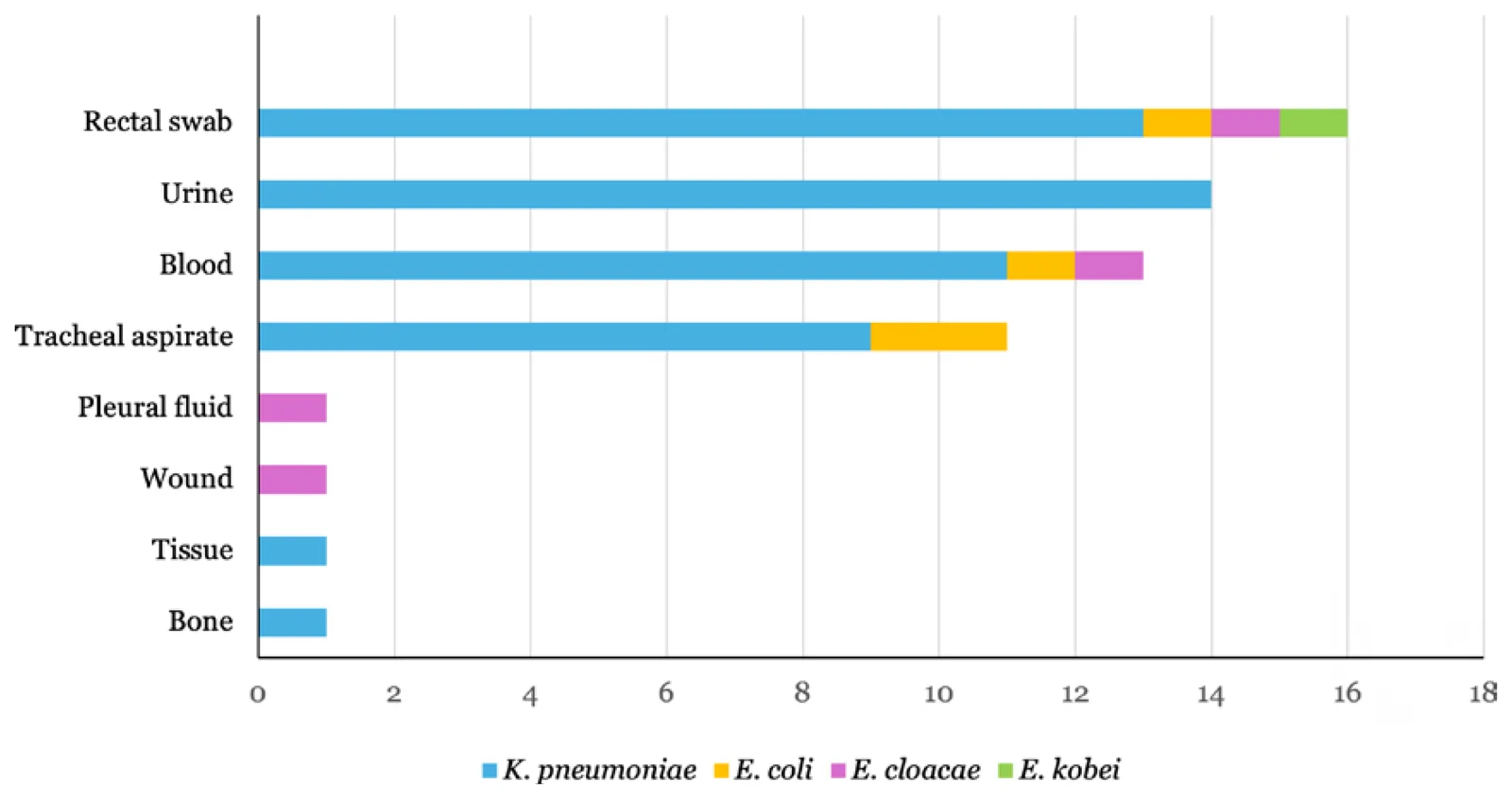
Distribution of Enterobacterales isolates according to the clinical samples

**Table 1:** Primers used to detect carbapenemase genes via multiplex PCR

Carbapenemase		Primer	Amplicon size	Reference
KPC	Forward	TGCAGAGCCCAGTGTCAGTTT	138 bp	Bhatt et al. (5)
	Reverse	CGCTCTATCGGCGATACCA		

NDM	Forward	GCAGCACACTTCCTATCTCG	202 bp	Ravikant et al. (6)
	Reverse	GTCCATACCGCCCATCTTGT		

OXA-48	Forward	GCGTGGTTAAGGATGAACAC	438 bp	Bhatt et al. (5)
	Reverse	CATCAAGTTCAACCCAACCG		

**Table 2:** Distribution of carbapenemases in each clinical sample according to rapid immunoassay and PCR

Isolate	Organism	Sample	mCIM	PCR Test	Rapid Test
1	*K. pneumoniae*	Rectal swab	POS	NDM	NDM
2	*E. cloacae*	Pleural fluid	POS	NDM	NDM
3	*E. cloacae*	Wound	NEG	NDM	Not detected
4	*E. coli*	TASP	NEG	Not detected	Not detected
5	*K. pneumoniae*	Blood	POS	NDM and KPC	NDM
6	*K. pneumoniae*	Blood	POS	KPC	Not detected
7	*K. pneumoniae*	Blood	POS	NDM and KPC	NDM
8	*K. pneumoniae*	TASP	POS	NDM and KPC	NDM
9	*K. pneumoniae*	Rectal swab	POS	NDM	NDM
10	*K. pneumoniae*	Rectal swab	POS	NDM	NDM
11	*K. pneumoniae*	Urine	POS	Not detected	Not detected
12	*K. pneumoniae*	Blood	POS	NDM and KPC	NDM
13	*K. pneumoniae*	Rectal swab	POS	NDM and OXA-48	NDM
14	*K. pneumoniae*	TASP	POS	NDM and KPC	NDM
15	*K. pneumoniae*	Blood	POS	NDM and KPC	NDM
16	*K. pneumoniae*	TASP	POS	NDM and KPC	NDM
17	*K. pneumoniae*	TASP	NEG	Not detected	Not detected
18	*K. pneumoniae*	Urine	POS	NDM and KPC	NDM
19	*K. pneumoniae*	TASP	POS	NDM and KPC	NDM
20	*K. pneumoniae*	Rectal swab	POS	NDM and KPC	NDM
21	*K. pneumoniae*	Urine	POS	Not detected	Not detected
22	*E. coli*	TASP	NEG	Not detected	Not detected
23	*K. pneumoniae*	Urine	POS	Not detected	NDM
24	*K. pneumoniae*	Urine	POS	Not detected	Not detected
25	*K. pneumoniae*	Blood	NEG	NDM	Not detected
26	*K. pneumoniae*	Blood	POS	OXA	Not detected
27	*K. pneumoniae*	TASP	POS	NDM and KPC	NDM
28	*E. coli*	Rectal swab	POS	NDM and KPC	NDM
29	*K. pneumoniae*	Urine	POS	NDM	NDM
30	*K. pneumoniae*	Rectal swab	POS	NDM	NDM
31	*K. pneumoniae*	Urine	NEG	Not detected	Not detected
32	*K. pneumoniae*	Blood	NEG	Not detected	Not detected
33	*E. cloacae*	Rectal swab	NEG	Not detected	Not detected
34	*K. pneumoniae*	Rectal swab	POS	NDM	NDM
35	*K. pneumoniae*	TASP	POS	Not detected	Not detected
36	*K. pneumoniae*	Blood	NEG	Not detected	Not detected
37	*K. pneumoniae*	Rectal swab	POS	Not detected	Not detected
38	*K. pneumoniae*	Rectal swab	POS	NDM	NDM
39	*K. pneumoniae*	Urine	POS	NDM	NDM
40	*K. pneumoniae*	Urine	POS	NDM	NDM
41	*K. pneumoniae*	Rectal swab	POS	NDM	NDM
42	*K. pneumoniae*	Rectal Swab	POS	NDM	NDM
43	*K. pneumoniae*	Urine	NEG	Not detected	Not detected
44	*K. pneumoniae*	Urine	POS	NDM	NDM
45	*K. pneumoniae*	Urine	NEG	Not detected	Not detected
46	*K. pneumoniae*	TASP	POS	NDM	NDM
47	*K. pneumoniae*	Rectal swab	POS	NDM and KPC	NDM
48	*K. pneumoniae*	Urine	NEG	Not detected	Not detected
49	*K. pneumoniae*	Blood	POS	NDM	NDM
50	*E. cloacae*	Blood	NEG	Not detected	Not detected
51	*K. pneumoniae*	Blood	POS	NDM and KPC	NDM
52	*E. coli*	Blood	POS	NDM and KPC	NDM
53	*K. pneumoniae*	Tissue	NEG	Not detected	Not detected
54	*K. pneumoniae*	Urine	POS	NDM	NDM
55	*K. pneumoniae*	Bone	NEG	Not detected	Not detected
56	*K. pneumoniae*	Rectal swab	POS	NDM and KPC	NDM
57	*K. pneumoniae*	TASP	POS	NDM and KPC	NDM
58	*E. kobei*	Rectal swab	POS	NDM and KPC	NDM

POS = positive; NEG = negative

**Table 3:** Comparison of the NG-CARBA-5 test result and multiplex PCR

	PCR-positive	PCR-negative	Total	Sensitivity (TP/TP + FN) × 100	89.7
NG-CARBA-5 test positive	35 (TP)	1 (FP)	36	Specificity (TN/TN + FP) × 100	94.7
NG-CARBA-5 test negative	4 (FN)	18 (TN)	22	PPV (TP/TP + FP) × 100	97.2
Total	39	19	58	NPV (TN/TN + FN) × 100	81.8
